# Development of a hepatic cryoinjury model to study liver regeneration

**DOI:** 10.1242/dev.203124

**Published:** 2024-07-31

**Authors:** Marcos Sande-Melon, David Bergemann, Miriam Fernández-Lajarín, Juan Manuel González-Rosa, Andrew G. Cox

**Affiliations:** ^1^ Peter MacCallum Cancer Centre, Melbourne, Victoria 3000, Australia; ^2^ The Sir Peter MacCallum Department of Oncology, The University of Melbourne, Melbourne, Victoria 3000, Australia; ^3^ Cardiovascular Research Centre, Massachusetts General Hospital Research Institute, Charlestown Navy Yard Campus, 149, 13th Street, MA 02129, USA; ^4^ Harvard Medical School, Boston, MA 02115, USA; ^5^ Biology Department, Morrissey College of Arts and Sciences, Boston College, Chestnut Hill, MA 02467, USA; ^6^ Department of Biochemistry and Pharmacology, The University of Melbourne, Melbourne, Victoria 3000, Australia

**Keywords:** Zebrafish, Liver regeneration, Cryoinjury, Inflammation, Proliferation, Fibrosis, Necrosis, Apoptosis

## Abstract

The liver is a remarkable organ that can regenerate in response to injury. Depending on the extent of injury, the liver can undergo compensatory hyperplasia or fibrosis. Despite decades of research, the molecular mechanisms underlying these processes are poorly understood. Here, we developed a new model to study liver regeneration based on cryoinjury. To visualise liver regeneration at cellular resolution, we adapted the CUBIC tissue-clearing approach. Hepatic cryoinjury induced a localised necrotic and apoptotic lesion characterised by inflammation and infiltration of innate immune cells. After this initial phase, we observed fibrosis, which resolved as regeneration re-established homeostasis in 30 days. Importantly, this approach enables the comparison of healthy and injured parenchyma within an individual animal, providing unique advantages to previous models. In summary, the hepatic cryoinjury model provides a fast and reproducible method for studying the cellular and molecular pathways underpinning fibrosis and liver regeneration.

## INTRODUCTION

Regeneration is defined as the anatomical regrowth of an injured organ to restore homeostatic functions. Unlike most human organs, the liver exhibits an unparalleled capacity to regenerate. The process of liver regeneration is multifaceted, as it requires a complex tissue comprising multiple cell types to sense the extent of the injury and mount an appropriate compensatory regrowth response (Michalopoulos and Bhushan, 2021). Because of this complexity, the molecular underpinnings of liver regeneration are poorly understood.

Chronic liver diseases, such as viral infection, alcohol and non-alcoholic steatohepatitis, often exhibit features of a maladaptive response to injury known as fibrosis (Pellicoro et al., 2014). The phenomenon of fibrosis is a common wound-healing response in nature in which resident fibroblasts deposit extracellular matrix. In most cases, fibrosis inhibits the regenerative response of organs. However, zebrafish can resolve fibrotic scars to enable organ regeneration (e.g. cardiac regeneration following myocardial infarction; González-Rosa et al., 2011; Schnabel et al., 2011; Chablais et al., 2011).

Several methods have been used to study liver regeneration in animal models. Among these methods, partial hepatectomy (PHx) is a surgical approach in which a liver lobe is resected, leaving the remnant liver lobes to regenerate (Mitchell and Willenbring, 2008; Kan et al., 2009; Sadler et al., 2007). In the PHx model of regeneration, inflammation or fibrosis has a limited role due to the absence of necrotic regions of tissue. The other dominant methods to study liver regeneration involve exposure to hepatotoxins that induce liver injury and, in some cases, fibrosis [e.g. CCl_4_ (Constandinou et al., 2005; Rubin et al., 1963; Iredale et al., 1996), DMN (Ala-Kokko et al., 1987; Kitamura et al., 2002; Yoshida et al., 2004), TAA (Müller et al., 1988; Salguero Palacios et al., 2008; Ding and Zhuo, 2013), DDC (Fickert et al., 2007), APAP (Mossanen and Tacke, 2015; Bhushan et al., 2014; Bhushan and Apte, 2019; North et al., 2010) and ethanol (Passeri et al., 2009; Popper and Leiber, 1980; Tsukamoto et al., 1986; Cordero-Espinoza and Huch, 2018)]. However, hepatotoxin-based approaches take months to develop liver injury and are therefore less amenable to rapid biological discovery. More recently, genetically encoded ablation models have been developed that enable specific liver cell types to be depleted [e.g. DTA (Sun et al., 2020) and NTR (Curado et al., 2008)]. Each model has unique advantages and disadvantages; however, collectively, they have led to remarkable advancements in our understanding of liver disease and regeneration.

Motivated to have a model of liver injury that is spatially localised yet retains hallmarks of inflammation and fibrosis, we sought to adopt the cryoinjury approach successfully deployed in the cardiac field (González-Rosa et al., 2011; Schnabel et al., 2011; Chablais et al., 2011). To this end, we describe here the development of a hepatic cryoinjury model of liver regeneration. We show that the hepatic cryoinjury model has no impact on survival, and reproducibly leads to the liver regenerating within 30 days. Importantly, we observe that cryoinjury triggers an inflammatory phase associated with the clearance of necrotic and fibrotic tissue, followed by subsequent proliferation of hepatocytes at the injury site. A significant advantage of this model is that it enables the comparative analysis of healthy and injured parenchyma in a single animal. We anticipate that this hepatic cryoinjury model will facilitate the discovery of the cellular and molecular underpinnings of fibrosis and regeneration.

## RESULTS

### Hepatic cryoinjury in adult zebrafish induces localised cell death

**Fig. 1.**
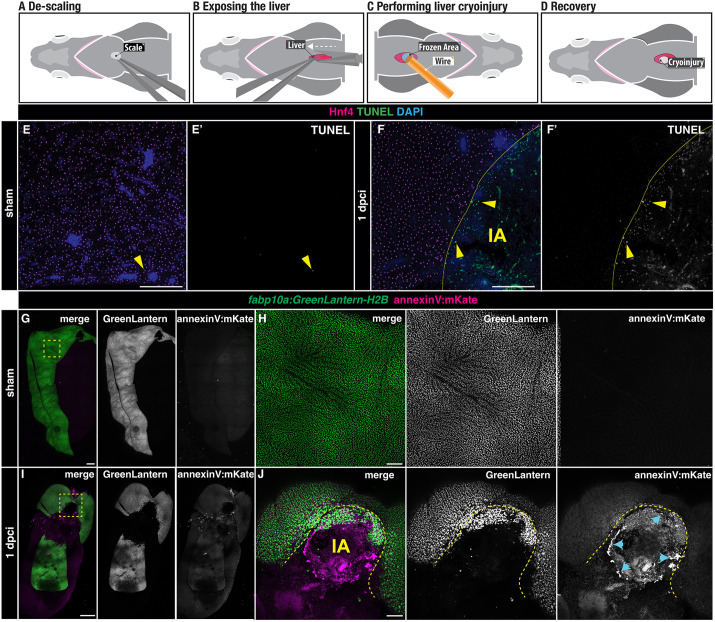
**Liver cell death following cryoinjury.** (A-D) Simplified schematics illustrating the cryoinjury procedure in the zebrafish liver. (A) The zebrafish liver is placed ventral side up to facilitate the surgery. (B) A small incision near the midline exposes the ventral liver lobe. (C) The frozen cryoprobe is applied to the liver surface for 15 s to induce injury. (D) The damaged area in the liver appears as a blister-like structure at 1 dpci. (E,F) TUNEL-staining of sham-operated (E,E′) and injured (F,F′) liver sections at 1 dpci. IA, injured area. Yellow arrowheads indicate TUNEL^+^ cells; yellow dashed lines indicate the border zone. (G-J) *Tg(fabp10a: GreenLantern-H2B; annexinV:mKate) in toto* acquisitions of sham-operated (G,H) and 1 dpci (I,J) livers. (I,J) The IA (yellow dashed line) is identifiable by the absence of GreenLantern-H2B^+^ hepatocytes. Blue arrowheads indicate AnnexinV-mKate^+^ cells. Scale bars: 500 µm.

We developed a novel method to study liver regeneration upon a localised cryoinjury of one of the liver lobes. Out of the three lobes present in the zebrafish liver, we chose the ventral lobe due to its surgical accessibility. In preparation for the surgery, zebrafish were immersed in anaesthetic and placed ventrally facing up in a foam holder (Fig. 1A). To create a reproducible and stereotypical injury, we performed all injuries at the level of the pectoral fins and toward the midline. First, a small incision was performed to expose the liver (Fig. 1B). Once the liver was visible, the excess water was gently removed using a rolled tissue. Then, the cryoinjury probe, which was cooled in liquid nitrogen for 1 min, was placed on the liver surface for 15 s until thawing (Fig. 1C). Fish were quickly transferred into a freshwater tank and reanimated by pipetting water into their gills for 30 s (Fig. 1D).

To assess the extent of cauterisation after cryoinjury, we employed TUNEL staining in liver sections of adult zebrafish with hepatocytes labelled for Hnf4, comparing sham and 1 day post-cryoinjury (dpci, Fig. 1E,F) livers. Sham-operated animals showed no TUNEL^+^ staining (Fig. 1E,E′). In contrast, cryoinjury caused an injured area (IA) comprised TUNEL^+^ cells surrounded by viable Hnf4^+^ hepatocytes (Fig. 1F,F′). To determine the extent to which this injury model induces apoptosis, we performed cryoinjury in *Tg*(*fabp10a:GreenLantern-H2B; actb2:annexinV*-*mKate*) adult zebrafish in which apoptotic cells will be labelled with the mKate^+^ signal (Fig. 1G-J). Although we did not detect apoptosis in sham-operated animals (Fig. 1G,H), AnnexinV-mKate^+^ apoptotic cells were observed around the injured area at 1 dpci, which can be easily detected by the localised loss of GreenLantern^+^ hepatocytes (Fig. 1I,J). Interestingly, the AnnexinV-mKate^+^ cells were distributed in an annular field around the injured area but not in the central region (Fig. 1J). Together, these results suggest that hepatic cryoinjury induces a localised cell death response that is characterized by a central necrotic core surrounded by a region of apoptotic cells at the injured border area.

### Liver regenerates upon cryoinjury in adult zebrafish

**Fig. 2.**
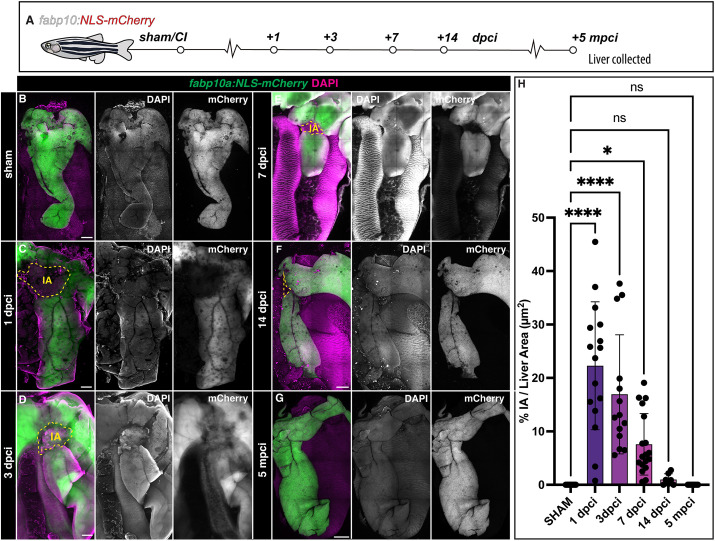
**Progression of liver regeneration after cryoinjury.** (A) A simplified schematic illustrating the collection of livers timeline after cryoinjury. (B-G) Whole-mount images of cleared zebrafish livers at the indicated stages of regeneration. Yellow dashed lines indicate the border zone. (H) Quantification of the IA area compared with the visible liver parenchyma area (*n*=16, 16, 14, 18, 7 and 12, left to right). Data are mean±s.d. *P*-values were calculated using one-way ANOVA followed by Tukey's multiple comparisons test (*P<0.05, ****P<0.0001). Scale bars: 500 µm.

To understand the temporal dynamics of liver regeneration upon cryoinjury, we examined the extent of recovery at different times post-cryoinjury using *Tg(fabp10a:NLS-mCherry)* zebrafish livers (Fig. 2A). Dissected livers were cleared, rendering them transparent, and subsequently scanned using *in toto* confocal imaging of the gastrointestinal block (Fig. 2B-G, Figs S1A-I and S2A-I).

Hepatic cryoinjury induced severe damage in the ventral lobe at 1 dpci, which was evident in histological sections and by the loss of the hepatocyte reporter fluorescence (Fig. 2B,C, Figs S2B,C and S3C-E). Despite the extent of the injury, ∼93% of the animals survived this surgical procedure (Fig. S2O; *n*=444). We observed a gradual recovery of mCherry^+^ hepatocyte expression from 3 dpci onwards, reducing the size of the injured area by confocal microscopy and histology (Fig. 2D, Figs S2D and S3F).

At 7 dpci, when other liver injury models have fully regenerated (Kan et al., 2009; Sadler et al., 2007; Bhushan et al., 2014; Benmoshe et al., 2022), the lesion is still clearly visible in cryoinjured livers (Fig. 2E, Figs S2E and S3H). The injured area is almost fully repaired at 14 dpci, as regeneration progressed (Fig. 2F and Fig. S2F). Prolonged follow-up at 21 dpci (Fig. S2G), 3 months post-cryoinjury (mpci; Fig. S2H), 5 mpci (Fig. 2G), 8 mpci (Fig. S3I), and 1 year post-cryoinjury (ypci; Fig. S2I) showed a fully regenerated liver parenchyma. Quantification of the injured area as a percentage of the total liver area at the different time points collected through this study indicates that the liver regenerates progressively over 2 weeks (Fig. 2H).

To determine whether sex or age are biological variables that modify the response of the liver to cryoinjury, we next compared the injury area in male and female zebrafish cryoinjured at either 4 or 9 months of age (Fig. S2J). Although we found that injuries in females tended to be smaller than those of males at 7 dpci, we did not find significant differences among groups (Fig. S2J-M,P).

Next, to ascertain whether pre-existing hepatocytes give rise to the regenerated hepatic parenchyma, we generated a hepatocyte-specific doxycycline-inducible Cre line for lineage tracing hepatocytes [Tg(*fabp10a:Tet-ON-Cre*)]. We treated Tg(*fabp10a:Tet-ON-Cre*; *ubb*:Switch) animals with doxycycline, which induced genetic labelling of virtually all hepatocytes before injury (Fig. S2Q,R). At 7 dpci, we observed recombined hepatocytes surrounding the injury area, suggesting that pre-existent hepatocytes contribute in part to the regeneration of the liver upon cryoinjury (Fig. S2S,T). Collectively, these results establish the hepatic cryoinjury model as a reproducible approach for inducing a spatially localised injury that is repaired over several weeks.

### Hepatic cryoinjury induces a transient fibrotic response

**Fig. 3.**
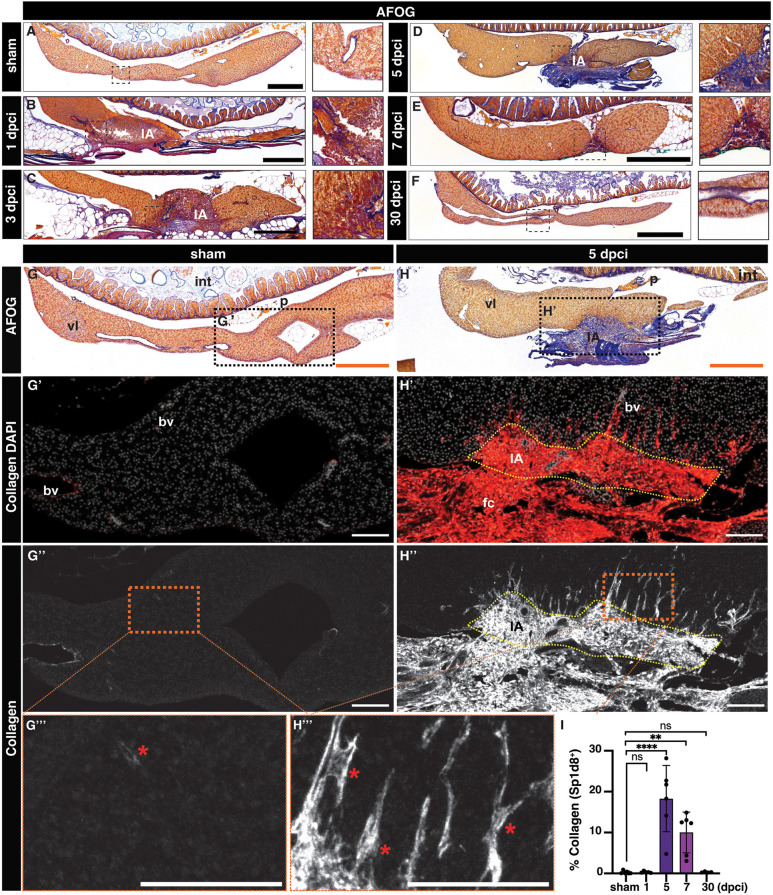
**Transient fibrotic deposition during liver regeneration after cryoinjury.** (A-H) AFOG staining in sections of representative sham-operated (A,G) or injured livers (B-F,H) at the indicated stages. Blue indicates collagen; red indicates cell debris and fibrin. Anterior is towards the left; dorsal is towards the top. Outlined areas are shown at higher magnification. (G′,H′) Adjacent sections from the samples showed in G and H, immunostained using an anti-Col1a1 antibody and counterstained with DAPI. Asterisks indicate Col1a1 deposition in the liver parenchyma. (G″-H‴). Pseudo-colored Col1a1 signal (white) from G’ and H’. Magnifications of the outlined areas are shown in G‴ and H‴. Red asterisks indicate Col1a1 accumulation. (I) Quantification of the collagen area of livers from the indicated cohorts, normalized to the liver parenchyma area (*n*=6, 5, 6, 6 and 4, left to right). Data are mean±s.d. *P*-values were calculated using one-way ANOVA followed by Tukey's multiple comparisons test (**P<0.01, ****P<0.0001). bv, blood vessel; fc, fibrotic cap; IA, injured area; int, intestine; p, pancreas; vl, ventral lobe. Scale bars: 100 µm (white); 500 µm (orange and black).

Fibrosis is an evolutionarily conserved mechanism of liver repair that is beneficial when transient but can be maladaptive in chronic liver disease (Pellicoro et al., 2014). Given the extent of cell death observed after cryoinjury, we wanted to determine whether this injury model involved a fibrotic response. To this end, we used the Fuchsin Orange G (AFOG) staining (Chablais et al., 2011; Mikami et al., 1975) to determine changes in ECM in response to cryoinjury. In the sham zebrafish liver, we mostly detected collagen surrounding the basement membrane of major blood vessels (Fig. 3A). At 1 dpci, the insulted liver tissue started to develop collagen and fibrin deposition at the IA (Fig. 3B), which gradually increased (Fig. 3C-E), reaching a fibrotic peak at 5 dpci (Fig. 3D,H and Fig. S3A,B). Immunofluorescence staining with a collagen type I-specific antibody revealed the presence of interstitial collagen depositions in the IA at 5 dpci (Fig. 3H-H‴), which is absent in sham-operated animals (Fig. 3G-G‴). During the next 2 weeks, fibrosis was gradually cleared and was almost undetectable at 30 dpci (Fig. 3F), when the structure of the liver parenchyma was fully restored. Quantification of the collagen staining confirmed a significant increase in collagen deposition at 5 and 7 dpci compared with sham-operated, 1 and 30 dpci livers (Fig. 3I). These results suggest that hepatic cryoinjury in the zebrafish leads to the formation of a transient fibrotic deposition, which is resolved within 30 days.

### Hepatic cryoinjury triggers a transient inflammatory response

**Fig. 4.**
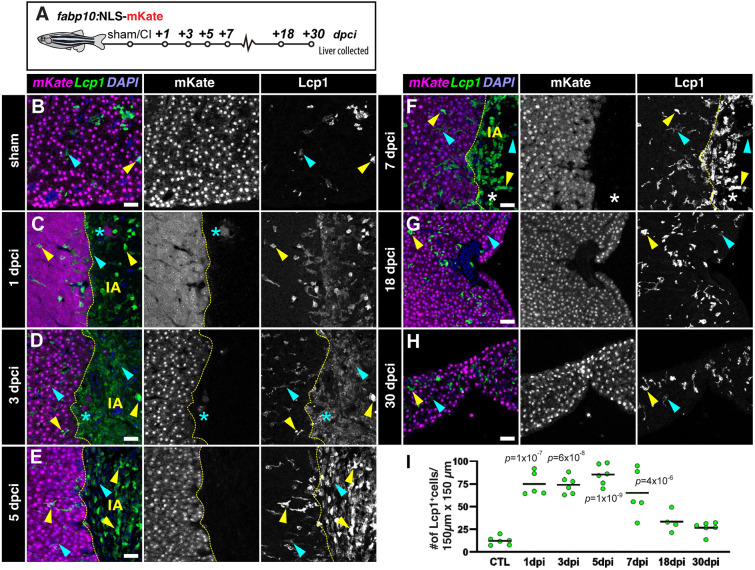
**Cryoinjury induces the local and transient infiltration of leukocytes.** (A) Schematic representation of the experiment workflow. (B-H) Sections of livers from *Tg(fabp10a:NLS-mKate)* animals at the indicated stages, immunostained to detect hepatocyte nuclei (mKate) and leukocytes (Lcp1). Cyan arrowheads indicate low signal Lcp1^+^cells; dashed yellow line indicates the border zone; IA, injured area; yellow arrowheads indicate high signal Lcp1^+^ cells; asterisks indicate spared hepatocytes surrounded by injured/necrotic tissue area. (I) Quantification of the number of Lcp1^+^ cells in designated regions (*n*=6, 5, 6, 6, 5, 4 and 6, left to right). Solid black line indicates the mean. *P*-values wee calculated using one-way ANOVA followed by Tukey's multiple comparisons test. Scale bars: 25 µm.

The initial phase of liver regeneration is often associated with inflammation, as leukocytes infiltrate the injured liver tissue (Pellicoro et al., 2014; Wang et al., 2021). Leukocytes are responsible for the phagocytosis of apoptotic and necrotic cells, and the secretion of cytokines that promote hepatocyte survival and proliferation during liver tissue repair. To assess whether hepatic cryoinjury caused inflammation, we stained liver sham-operated and injured sections of *Tg*(*fabp10a*:NLS-mKate) zebrafish, carrying hepatocyte-specific mKate^+^ nuclear transgene expression in combination with the pan-leukocyte marker L-plastin (Lcp1) antibody (Fig. 4A). In homeostatic livers, we observed a few Lcp1^+^ leukocytes in the liver tissue (Fig. 4B). However, we detected a rapid infiltration of inflammatory cells after injury at 1 and 3 dpci, both in the healthy parenchyma and the cryoinjured area (Fig. 4C,D). Immune infiltration was maintained for 7 days post-injury in the healthy liver parenchyma and the insulted area (Fig. 4E,F). The presence of leukocytes gradually decreased at 18 dpci (Fig. 4G), and reached basal levels at 30 dpci (Fig. 4H), when the damaged area was no longer detected. Analysis and quantification of sham-operated and injured liver sections at different timepoints with Lcp1 staining showed a significant increase in the number of infiltrated leukocytes at 1, 3, 5 and 7 dpci (Fig. 4I).

Using Lcp1, we gained some insights into the dynamics of the inflammatory response, but we lacked granular information about the specific populations and their potential role during regeneration. To characterize this process further, we stained sections from sham-operated and injured livers with antibodies that recognize Mpx, which is a neutrophil-specific marker (Fig. S4A). Although we did not find neutrophils in homeostatic livers (Fig. S4B,B′), we detected infiltrated neutrophils at 1, 3 and 5 dpci specifically in the cryoinjured area but not in the contralateral lobe (Fig. S4C-E′). This response was transient, as analysis of livers at 7 dpci revealed a reduction to basal levels by that stage (Fig. S4F,F′).

Macrophages are essential for regeneration of the zebrafish heart, fin and brain (Simões and Riley, 2022; Kyritsis et al., 2012; Laplace-Builhé et al., 2021). To study whether macrophages are necessary for liver regeneration upon cryoinjury, we performed intraperitoneal (IP) injections of either PBS or clodronate liposomes to deplete macrophages during the regenerative process. Adult livers were collected to assess the injured area and were immunostained to detect Mfap4^+^ macrophages at 3 and 7 dpci (Fig. S5A). We did not find significant differences in the regenerative response in livers treated with clodronate liposomes (Fig. S5B,C and S5J), albeit there was a noticeable reduction in Mfap4^+^ cells in the treated group (Fig. S5F-I). Collectively, these experiments confirm that hepatic cryoinjury is associated with the local and transient infiltration of leukocytes into the injured area.

### Cryoinjury induces both localised and distal compensatory hyperplasia

**Fig. 5.**
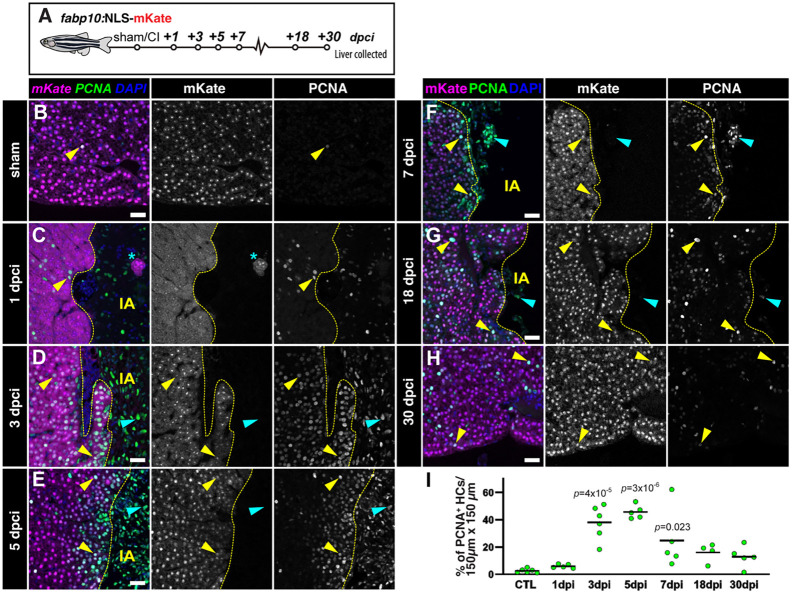
**Local hepatocyte hyperplasia upon cryoinjury.** (A) Schematic representation of the experiment workflow. (B-H) Liver sections from *Tg(fabp10a:NLS-mKate)* animals at the indicated stages, immunostained to detect proliferation (PCNA) and hepatocyte nuclei (mKate). Yellow arrowheads indicate proliferating hepatocytes. Cyan arrowheads indicate other cell types actively cycling. Dashed yellow line indicates the separation between healthy and injured liver parenchyma; IA, injured area; asterisks indicate spared hepatocytes surrounded by injured/necrotic tissue area. (I) Hepatocyte proliferation index in the border zone at the indicated stages (*n*=6, 5, 6, 5, 5, 4 and 5, left to right). Solid black line indicates the mean. *P*-values were calculated using one-way ANOVA followed by Tukey's multiple comparisons test. Scale bars: 25 µm.

Two regenerative mechanisms could be at play in the cryoinjury model, namely compensatory hyperplasia, in which hepatocyte proliferation occurs throughout the parenchyma until liver mass reaches homeostasis, or epimorphic regeneration, in which hepatocytes proliferate in a spatially localised region around the injury. To understand the regenerative response upon hepatic cryoinjury, we examined hepatocyte proliferation. To identify proliferative hepatocytes, we stained sections of *Tg(fabp10a:NLS-mKate)* with the proliferating cell nuclear antigen (PCNA; Fig. 5A) antibody. Additionally, we performed BrdU pulse-labelling studies, injecting BrdU in sham and injured zebrafish at 2 dpci (Fig. S6A). Quantification of hepatocyte proliferation (mKate^+^/PCNA^+^) confirmed that there were few mKate^+^/PCNA^+^ cells (Fig. 5B,I) and mCherry^+^/BrdU^+^ hepatocytes (Fig. S6B,D,E) in sham-operated animals, consistent with the slow turnover of hepatocytes in adulthood during homeostasis (Heinke et al., 2022; VanSaun et al., 2013; Arrojo e Drigo et al., 2019). In contrast, proliferating hepatocytes were significantly more abundant around the injured area during the first week of recovery after cryoinjury (Fig. 5C-F and Fig. S6C-K).

Hepatocyte proliferation peaked between 3 and 7 dpci, which correlates with the timing of reduction in the IA (Fig. 5I). Analysis of proliferating hepatocytes shows a localised hyperplasia, with a significantly higher density of cycling cells in areas surrounding the injured area compared with the contralateral lobes (Fig. S6D-F,I-K). This recovery of hepatocytes around the cryoinjured area is consistent with an epimorphic regeneration. Notably, we detected proliferating hepatocytes in the contralateral lobe (Fig. S6C-K), indicative of an adaptive compensatory hyperplasia. Hepatocyte proliferation decreased during later stages of regeneration to a baseline at 30 dpci (Fig. 5G,H). Therefore, our data suggest that hepatic cryoinjury stimulates features of both epimorphic and compensatory hyperplasia during liver regeneration.

### The biliary and endothelial network is re-established during regeneration

**Fig. 6.**
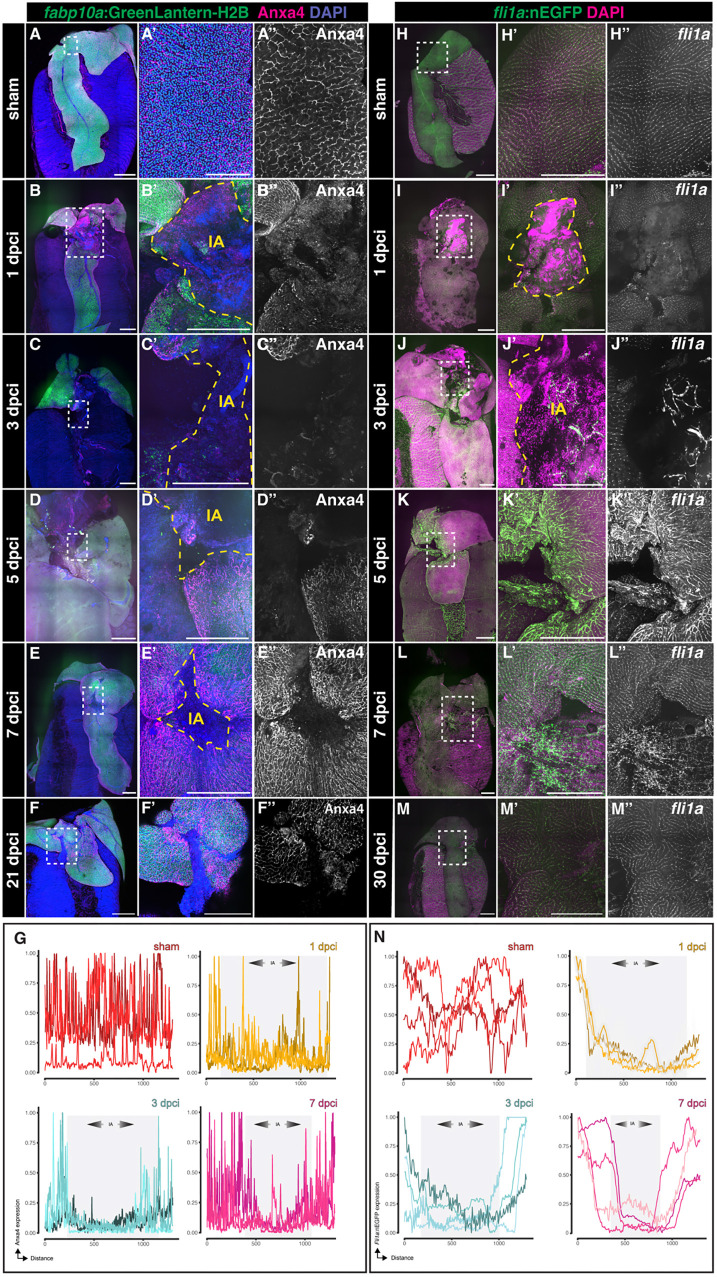
**Biliary epithelial cells (BECs) and endothelial cells (ECs) recover upon cryoinjury.** (A-F″) Whole-mount imaging of *GreenLantern*^+^ hepatocytes and Anxa4^+^ biliary epithelial cells from sham-operated (A-A″) or injured (B-F″) livers at the indicated stages. The outlined areas are shown at higher magnification. (G) Mean intensity profile of Anxa4 within the IA at the designated stages is represented by individual lines for each sample, with the IA delineated in grey (*n*=4 throughout). (H-M″) Whole-mount acquisitions of GFP^+^ ECs from sham-operated (H-H″) or injured (I-M″) livers at the indicated stages. The outlined areas are shown at higher magnification. (N) Mean intensity profile of *fli1a*:nGFP within the IA at the designated stages is represented by individual lines for each sample, with the IA delineated in grey (*n*=4 throughout). Dashed yellow line indicates the border zone of the injured area; IA, injured area. Scale bars: 500 µm.

Adaptive liver regeneration that recovers hepatic function requires appropriate restoration of the biliary and vascular network, which comprise biliary epithelial cells (BECs) and endothelial cells (ECs), respectively. To examine whether BECs regenerate after hepatic cryoinjury, we collected *Tg(fabp10a:GreenLantern-H2B*) livers at 1, 3, 7 and 21 dpci (Fig. 6A-F and Fig. S7A-C), performed CUBIC clearing and immunodetected the BEC marker Annexin A4 (Anxa4). Anxa4^+^ BECs were detectable throughout the liver parenchyma in sham livers (Fig. 6A-A″).

However, the distribution of Anxa4^+^ BECs was disrupted in the IA at 1 and 3 dpci, indicating that bile duct structures were affected during this procedure (Fig. 6B-C,G, and Fig. S7C). A progressive recovery of the biliary network was observed at 7 and 21 dpci, as the insult area gradually reduced in size (Fig. 6E,F and Fig. S7C). Importantly, no differences were observed in the architecture of the biliary network in regenerated livers at 21 dpci compared with sham-operated animals (Fig. 6A″,F″), consistent with the regenerative response in hepatocytes. We documented a dynamic and significant increase in the proliferation of BECs after injury, peaking at 3 dpci (Fig. S6L-O). We also detected a modest but significant increase in BEC proliferation in the contralateral lobe at 3 dpci (Fig. S6P), suggesting that the regeneration of the biliary tree is predominantly driven by localized hyperplasia and not by compensatory growth.

To determine the dynamics of regeneration of the ECs after injury, we collected *Tg(fli1a:NLS-eGFP)* livers at 1, 3, 7 and 30 dpci (Fig. 6F-J and Fig. S6F-G). eGFP^+^ ECs were observed equally through the sham liver parenchyma (Fig. 6F-F″ and Fig. S6F,G). However, the distribution of ECs was affected in the IA at 1 dpci (Fig. 6G-G″ and Fig. S6F,G). Vascular recovery starts to be observed at 3 dpci, with branching morphogenesis of eGFP^+^ ECs appearing within the IA (Fig. 6H-H″ and Fig. S6F,G) at early stages, consistent with previous reports (Heinke et al., 2022). Interestingly, eGFP^+^ ECs are detected throughout the entire IA at 7 dpci, revealing that vasculature regeneration precedes hepatocyte and BEC regeneration (Fig. 6I-I″ and Fig. S6F,G). However, the endothelium appeared significantly disorganized in the IA compared with sham-operated animals (Fig. 6I″,F″), and this abnormal distribution persisted at 30 dpci (Fig. 6J-J″), even when the liver parenchyma was fully recovered. These findings demonstrate that hepatic cryoinjury disrupts the biliary and vasculature network, which is subsequently re-established during liver regeneration.

### Characterization of the transcriptional landscape after cryoinjury

**Fig. 7.**
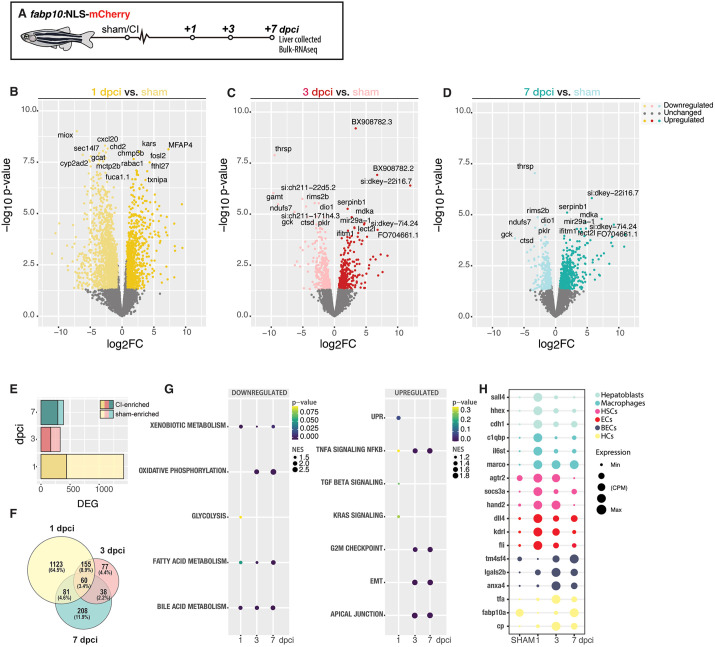
**Transcriptional signatures of liver regeneration upon cryoinjury.** (A) Schematic representation of the experiment workflow. (B-D) Volcano plots representing the comparison of 1, 3 and 7 dpci with sham-operated adult zebrafish. DEGs: FC ≥1.5 (darker dots) or ≤−1.5 (lighter dots); *P*≤0.05; with top DEG annotated. (E) Bar plot representing the number of upregulated and downregulated DEGs at 1, 3 and 7 dpci. (F) Venn diagram representing DEGs at 1, 3 and 7 dpci. (G) GSEA of liver cell types during liver regeneration. (H) Dotplot representing the expression of key genes for specific liver cell populations upon cryoinjury.

To study the transcriptional changes that occur during liver regeneration, we performed bulk RNA-seq on the injured region in the ventral lobe and contralateral lobe of livers dissected from *Tg(fabp10a:NLS-mCherry)* animals after sham-operation, 1, 3 and 7 dpci (Fig. 7A, Fig. S8A,B and Table S1). At 1 dpci, we detected 438 upregulated differentially expressed genes (DEGs) and 981 downregulated genes when compared with sham-control liver samples (Fig. 7B,E,F).

Gene Set Enrichment analysis (GSEA) identified the TNFα signalling [*P*-value, 0.318; normalised enrichment score (NES), −1.19] and TGF-β signalling (*P*-value, 0.235; NES, −1.16), consistent with the inflammatory and fibrotic features exhibited after cryoinjury (Fig. 7G). We also identified the unfolded protein response pathway (*P*-value, 0.074; NES, −1.40), a well-established component of the integrated stress response, as among the most enriched signatures after cryoinjury (Fig. 7G and Fig. S8H). We compared the expression levels of *atf4*, an effector transcription factor from the unfolded protein response, during development and adulthood (Fig. S9A). We could observe a significant upregulation of *atf4* and *atf3* specifically upon injury at 1 dpci, compared with homeostatic conditions during development (7 dpf) or sham-operated animals (Fig. S9B). *atf4* mRNA HCR staining revealed low *atf4* expression levels in sham controls (Fig. S9C) and an increase in its expression at the IA and injured border area at 1 dpci (Fig. S9D). Atf4 downstream targets involved in amino acid synthesis, transport and RNA charging were significantly upregulated at 1 dpci (Fig. S9E) (VanSaun et al., 2013). These results show a plausible previously unreported molecular target to modulate liver regeneration upon injury.

Many of the downregulated hallmarks and Gene Ontology (GO) enrichment (Fig. 7G and Fig. S8G), which we observed after cryoinjury at 1 dpci, are associated with housekeeping functions of hepatocytes, such as bile acid metabolism (*P*-value, 0.02; NES, 1.87), xenobiotic metabolism (*P*-value, 0.037; NES, 1.75) and fatty acid metabolism (*P*-value, 0.05; NES, 1.65) (Fig. 7G). At 3 dpci, 167 upregulated DEGs were detected, whereas 163 DEGs were downregulated (Fig. 7C,E,F) compared with the sham. GSEA of DEGs at 3 dpci revealed several hallmarks related to cell proliferation and migration, including the G2/M checkpoint signature (*P*<0.001; NES, −1.55) and epithelial-mesenchymal transition signature (EMT; *P*<0.001; NES, −1.76), respectively (Fig. 7G). These signatures are consistent with the timing of proliferation described after cryoinjury (Fig. 5D), and the increased inflammation and recruitment of leukocytes to the injured area in livers between 3 dpci and 7 dpci (Fig. 4D-F and Fig. S4D-F). To characterise changes in tissue composition after cryoinjury, we used cell-type specific genes to identify the dynamics of liver regeneration on the different cell types present in the liver during the first 7 dpci (Fig. 7B-D,H). At 1 and 3 dpci, we observed an increase in genes specifically expressed in ECs (Fig. 7H), which may be indicative of a quick angiogenesis response, as previously observed by confocal microscopy (Fig. 6I-J), quickly reducing by 7 dpci when the EC population seems almost fully recovered (Fig. 6L,N). At 1 dpci, we detected an enrichment in genes expressed in hepatoblast, which may explain the dedifferentiation of hepatocytes to a hepatoblast-like state (Fig. 7H). Genes associated with immune cells were enriched at 1 dpci upon injury, consistent with the early recruitment of immune cells towards the site of injury (Fig. 7H). At 3 dpci, we detected enrichment in genes specifically expressed by BECs, suggesting an active restoration of the biliary network (Fig. 7H). We detected an increase of hepatocyte transcripts, showing the initial recovery of the main liver parenchyma. HSCs were enriched at 3 dpci. This may explain the rapid development of the fibrotic response upon cryoinjury (Fig. 7H). By 7 dpci, we detected 97 upregulated and 290 downregulated DEGs compared with sham-operated livers (Fig. 7D-F), suggesting a gradual shift towards homeostasis in the liver. When we compared the expression between the injured area in the ventral lobe with the contralateral lobe, we observed a much bigger proportion of DEGs in the injured area when compared with the contralateral lobes (Fig. S8C-F). This might explain previous microscopy observations of a strong localised regeneration combined with a compensatory response in the livers during regeneration. In light of these studies, we conclude that the transcriptional landscape dynamically changes during regeneration as the liver responds to cryoinjury, undergoes regenerative growth and eventually returns to homeostasis.

## DISCUSSION

**Fig. 8.**
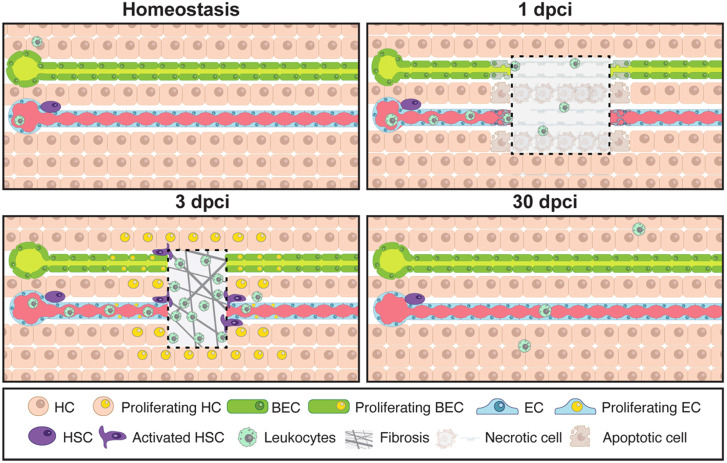
**Model of liver regeneration upon cryoinjury.** Schematic representation of the cellular events taking place upon cryoinjury during liver repair in the zebrafish.

Here, we introduce the hepatic cryoinjury model, which serves as a rapid and reliable method to investigate liver regeneration in a spatially localised manner (Fig. 8 and Table 1). The contact with the liver surface of the frozen cryoprobe generates a necrotic region, surrounded by apoptosis in the injured border area, resembling clinical liver injuries (VanSaun et al., 2013). Furthermore, necrotic cells trigger a robust innate immunological response and immune infiltration at the injury site. The hepatic cryoinjury model offers several advantages compared with the other injury models (Table 1). First, it creates a distinct localized injury, enabling the examination of injured and uninjured hepatic parenchyma in the same animal. Moreover, hepatic cryoinjury is easy to perform and visualise macroscopically (blister formation), which facilitates the investigation of the tissue regenerative microenvironment and the use of state-of-the-art sequencing and imaging techniques. Second, the cryoinjury model gives rise to necrotic and apoptotic tissue, and the localised infiltration of inflammatory cells, which are absent in other surgical models (i.e. HPx). Third, the hepatic cryoinjury model induces a transient fibrotic response not seen in other models, which resolves within 30 dpci without hindering liver repair. For these reasons, we anticipate that this unique model will aid in the discovery of the molecular mechanisms of fibrosis and regeneration.

**Table 1.**
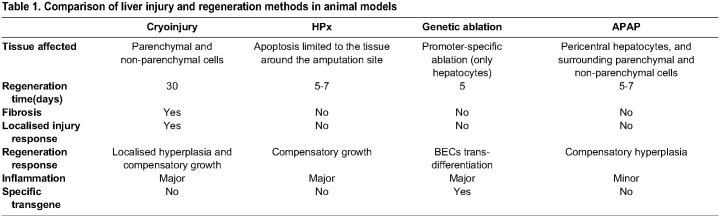
Comparison of liver injury and regeneration methods in animal models

Although the HPx model is almost 90 years old, it remains the gold standard of models for studying liver regeneration. Liver regeneration in rodent HPx models occurs rapidly via compensatory hyperplasia, in which hepatocyte proliferation peaks at 36 and 48 h post resection, before receding by 3 days, and liver homeostasis is re-established at 7 days (Michalopoulos and Bhushan, 2021; Arrojo e Drigo et al., 2019). Zebrafish HPx models show similar dynamics of hepatocyte proliferation with almost no detectable proliferative beyond 4 days (Kan et al., 2009; Sadler et al., 2007). A major point of difference between the HPx models and hepatic cryoinjury is the more prolonged time course of regeneration (30 days). We suspect this is partly due to transient fibrosis and the need to resolve damaged tissue before mounting a proliferative phase of regeneration.

Acetaminophen (APAP) overdose is the most common cause of acute liver failure (ALF), as it induces necrosis of pericentral hepatocytes, leading to a necrotic area in zone 3 of the liver parenchyma (Bhushan and Apte, 2019). The APAP model shares features of the hepatic cryoinjury model, such as the induction of region-specific necrosis (Table 1). However, one major distinction is that, owing to its mechanism of action, APAP targets pericentral hepatocytes, whereas the hepatic cryoinjury affects all cells present at the site of injury. Tissue loss due to APAP exposure is recovered by hepatocyte proliferation within 4 days, peaking at 2 days post APAP exposure (Benmoshe et al., 2022), which is similar to the kinetics of hepatocyte proliferation observed after HPx but earlier than we have observed after hepatic cryoinjury. Interestingly, hepatocyte proliferation after APAP exposure occurs in areas where healthy hepatocytes are in contact with necrotic tissue, rather than via compensatory hyperplasia (Michalopoulos and DeFrances, 1997), which is reminiscent of the epimorphic proliferative response observed after cryoinjury. Another similarity between cryoinjury and APAP is that APAP exposure triggers the infiltration of immune cells, activating an innate and adaptive immunological response (Bhushan and Apte, 2019). Given the crucial role of immunity in liver disease, it is beneficial that the hepatic cryoinjury model exhibits classic hallmarks of inflammation.

Genetic ablation models provide an invaluable approach for targeted cell ablation in animal models, elucidating the roles of various cell populations in liver tissue repair and regeneration (Torrence et al., 2021; Brazovskaja et al., 2024) (Table 1). These models have significantly contributed to our understanding of transdifferentiation events between BECs and hepatocytes in the context of tissue regeneration. Although the ability to selectively target specific cell populations presents clear advantages, generating cell type-specific promoter transgenic lines can be labour intensive and time consuming. In contrast to the cryoinjury model, which affects all parenchymal and non-parenchymal cells in the liver, cell-specific ablation techniques uniquely target distinct liver cell populations, thereby only allowing the investigation of the function of a unique liver cell of interest.

Liver fibrosis is a crucial feature in the development of chronic liver diseases and the progression towards hepatocellular carcinoma (HCC). Hepatic cryoinjury leads to a transient fibrotic response (peaking at 5 dpci) that was resolved within 30 dpci. Compared with other models of liver fibrosis (e.g. CCl_4_, TTA, ethanol or DDC), which typically develop over months (Cordero-Espinoza and Huch, 2018) (Table 1), fibrosis in the cryoinjury model occurs rapidly and is resolved over time. In future studies, the cryoinjury model could be used to explore the clonality of fibrosis and regeneration. To this end, recent advances in DNA-barcoding lineage-tracing methods (Weng et al., 2015; Taub, 2004; Yanger et al., 2014; Kramann et al., 2015; Shin et al., 2015) and spatial transcriptomics (McKenna et al., 2016; Raj et al., 2018) make it feasible to use the cryoinjury model to identify the mechanisms driving the resolution of fibrosis. This enhanced understanding is crucial for identifying innovative therapeutic strategies that promote liver repair and for combating fibrotic progression.

In summary, we have described a new hepatic cryoinjury and regeneration model that recapitulates key features of liver disease (Fig. 8). Given the rapid and reproducible nature of the technique, we suspect the model will offer opportunities to uncover the molecular underpinnings of liver regeneration.

## MATERIALS AND METHODS

### Experimental model and details

Experiments were conducted with adult zebrafish (*Danio rerio*) aged 4-9 months raised at a maximal 5 fish/l and maintained under the same environmental conditions: 27.5-28°C, 650-700 ms/cm, pH 7.5. The lighting conditions were 14:10 h (light: darkness) and 10% of water exchange a day. Experiments were approved by the Peter MacCallum Cancer Centre AEEC (E665) and the Institutional Animal Care and Use Committees of Massachusetts General Hospital and Boston College. All animal procedures conformed with the Prevention of Cruelty to Animals Act, 1986 (the Act), associated Regulations (2019) and the Australian code for the care and use of animals for scientific purposes, 8th edition, 2013 (the Code). The following transgenic zebrafish lines were used in this study: *Tg(fabp10a:NLS-mCherry)* (Mudbhary et al., 2014), *Tg(fabp10a:GreenLantern-H2B)^uom306^* (Campbell et al., 2020; Tan et al., 2024) and *Tg(fabp10a:rtTA;TRE-Cre)^uom307^*, herein referred to as *Tg(fabp10a:Tet-ON-Cre)*, *Tg(fabp10a:NLS-mKate)^bcz102^* and *Tg(fli1a:nEGFP)^y7^*, respectively (Lawson and Weinstein, 2002).

### Cryoinjury and analysis of the injured area

Fish are immersed in Tricaine (0.032%; wt/vol; Sigma) before surgery and placed in a foam holder with the ventral area facing up. Under a stereo microscope, scales above the liver area were removed using sharp forceps. Once the area had been cleaned, a small incision was made parallel to the skin using micro-dissecting scissors towards the midline, and the anterior fin was moved to expose the liver through the ventral zebrafish body. Once the liver is exposed, the excess water is removed using tissue paper (KimWipe). The cryoinjury probe of 1 mm diameter (Jaycar; Australia), which was cooled in liquid nitrogen for 1 min, was placed on the liver surface for 15 s until thawing. Sham operations consisted of placing the thawed cryoprobe on the exposed liver at room temperature. Fish are quickly placed in a freshwater tank and reanimated by pipetting water into their gills for 30 s. The complete surgical procedure takes 3-5 min per fish. Healthy fish should be swimming normally after 5 min. Fish are transferred to the normal tanks and put into their racks 10 min after surgery with a gentle water flow. The damaged tissue was easily identified either from the lack of fluorescence when using a hepatocyte-specific promoter line, which expresses a nuclear mCherry or GreenLantern signal specifically in hepatocytes (HCs) or from a blister-like injury in the liver surface. Injured and sham-injured livers were phenotyped, and acquisitions were performed using a Leica M165FC fluorescent stereomicroscope. The injured area was measured on Imaris Software 9.0 (Bitplane). Statistical analysis and graphs were generated using GraphPad Prism v9.

### Whole-mount CUBIC liver immunofluorescence and image processing

*In toto* immunofluorescence was performed as described previously (Kong et al., 2020). Animals were euthanized at different times post-injury by immersion in 0.16% Tricaine. Livers were collected and fixed in PFA 4% overnight at 4°C in rocking agitation. We washed the livers three times in PBS Tw 0.1% before transferring them to CUBIC-L (Matsumoto et al., 2019) for 18 h at 37°C. After CUBIC-L incubation, livers were washed in 1×PBS three times for 15 min. Livers were incubated overnight with blocking solution (5% BSA, 5% goat serum and 0.1% Tween-20). Primary and secondary antibodies were incubated for 1 day at 4°C in rocking agitation. Livers were transferred to CUBIC-R^+^ for 2 h at room temperature and embedded in CUBIC-R^+^-Agarose for imaging. Livers were mounted on a glass-bottom culture dish (MatTek Corporation) for confocal acquisition. Whole liver acquisitions were obtained with Zeiss LSM 780 and Nikon Sora Spinning-Disk confocal microscopes using 10× dry and 20× dry lenses. Images were acquired at 512×512 or 1024×1024 resolution. For every liver acquisition, tile-scan and *z*-stack modules were used to acquire a representative area of the liver. The injured area of the liver in whole-mount acquisitions was analysed and measured on Imaris Software 9.0 (Bitplane) and Fiji (Bowling et al., 2020). Acquisitions were saved as TIFF or JPEG on Fiji of the original files.

### BrdU^+^ and PCNA^+^ hepatocytes image analysis

Adult zebrafish were injected intraperitoneally at 2 dpci with 20 μl of 2.5 mg/ml of 5-bromo-2-deoxyuridine (BrdU, B5002-1G, Sigma). Livers were collected and processed for analysis at 5 dpci. *In toto* immunofluorescence was performed as described previously (Kong et al., 2020). Whole-mount immunofluorescence was performed using anti-BrdU [AB_2313786, Abcam, ab6326 BU1/75 (ICR1), rat, 1:150], anti-RFP (AB_945213, Abcam, ab62341, rabbit, 1:200), goat anti-rat IgG (H+L) Cross-Adsorbed Alexa Fluor 647 (ThermoScientific, A-21247, 1:300) and goat anti-rabbit IgG (H+L) Alexa Fluor 594 Antibody (Invitrogen, A-11012, 1:300). Using Imaris Software 9.0 (Bitplane), we used the co-localisation channel tool to create a new channel, which represents BrdU^+^ HCs. A spot segmentation of BrdU^+^ HCs and BrdU^−^ HCs was created. Manually, the site of injury was created as a surface. We used the new injured area surface and the ImarisXT/Distance-Transformation package (outside surface) to define the distance of every BrdU^+^ HC spot to the injury site. This new channel was masked into the BrdU^+^ HC spots, which were previously created. The new BrdU^+^ HC spots channel contains the specific distance to the injured area from every proliferating HC, named proliferating HC distance channel. A new spot segmentation was carried out using the proliferating HC distance channel. We exported the positions X, Y, Z and proliferating HC distance channel mean intensity from the last proliferating HC distance channel. Using R, the different datasets were selected to create a representative graph, using the ggplot2 package, of the presence of BrdU^+^ HCs or PCNA^+^ HCs towards the site of injury. The location of proliferating HCs was measured relative to the injured area, which was used as a point of origin.

### Clodronate intraperitoneal injections

Intraperitoneal (IP) injections of 10 μl PBS and clodronate liposomes (5 mg/ml) (C-005, Liposoma) were performed in each fish, 1 day before the cryoinjury in the adult zebrafish livers. Livers were collected with the rest of the gastrointestinal tract (GIT) and processed for analysis at 3 and 7 dpci. PBS and clodronate-injected livers were phenotyped and acquired using a Leica M165FC fluorescent stereomicroscope. The injured area was measured using Fiji Image Analysis Software (Bowling et al., 2020). Statistical analysis and graphs were generated using GraphPad Prism v9. Livers were fixed overnight in 4% PFA for cryosectioning and immunostaining.

### Doxycycline treatment

Adult fish were treated by immersion in doxycycline (30 mg/ml; Sigma D5207) for 72 h. Animals were kept in darkness at 28°C for the duration of the treatment. Animals were fed three times in clean fish water and placed back in the doxycycline-treated water.

### Zebrafish liver fixation and histological processing

To obtain histology sections for AFOG or immunofluorescence, adult zebrafish livers were collected with the rest of the GIT. This procedure minimized disruption of the fragile liver tissue. Adult zebrafish were euthanized by immersion in 0.16% tricaine and placed in ice-cold PBS. A ventral incision through the skin, muscle and peritoneal cavity was made, starting at the anus towards the pericardiac cavity. To avoid disrupting adhesions and scar tissue at the injury zone in early injury time points (1-7 dpci), the tissue adhering to the liver injury was left intact on the liver by performing an incision surrounding it. The GIT was detached at its anal part and slowly lifted, while cutting the vasculature connecting the dorsal part of the GIT to the peritoneum and gonads. Once completely lifted from the cavity, a final cut was made anteriorly on the oesophagus to extract the GIT. Samples were fixed overnight at 4°C in 10% neutral buffered formalin (HT501125, Sigma) and embedded in paraffin following standard processing (45 min incubations in ethanol 70%, 90%, 95%, 2×100%, 2×xylenes, and 4× paraffin washes). Sagittal sections (7 µm) were mounted on SuperFrost+ slides (12-550-15, Fisherbrand) and dried overnight at 37°C before storage or processing.

### Acid Fuchsin Orange G staining and imaging

For Acid Fuchsin Orange G (AFOG) staining, sections were dewaxed and rehydrated using standard procedures and then stained as described previously (Koth et al., 2020). Collagen was stained blue, while fibrin and cell debris appeared red. Sections were imaged on a Leica DM6-B upright microscope equipped with a Leica DFC7000 camera using the 10× or 20× objectives. Whole GIT images were acquired using the automated navigator and stitching function in LAS-X software.

### Hematoxylin and Eosin staining and imaging

For Hematoxylin and Eosin (H&E) staining, sections were sectioned at 5 μm. Slides were dewaxed and rehydrated using standard procedures. Slides were stained with Mayer's Hematoxylin solution (ab220365; Abcam) for 5 min. The nuclear staining was differentiated for 15 s in 0.37% HCl prepared in 70% ethanol, and the slides were washed in tap water for 10 min. Sections were incubated on 0.1% Eosin Y solution (ab246824; Abcam) for 3 min in 0.1% water with acetic acid. Sections were washed in double-distilled water. The sections were dehydrated in a water/ethanol series, cleared in xylol and mounted in DPX mounting medium (06522; Sigma-Aldrich). Sections were imaged on an Olympus SLIDEVIEW VS200 automated slide scanner using 10×, 20×, 40× and 63× lenses. Whole GIT sections were acquired, and images were analysed using QuPath (Bankhead et al., 2017) and Olympus LS software.

### Immunofluorescence in cryosections

Cryosections were incubated for 30 min in PBS at 37°C. Sections were washed three times on PBST (0.1% Tween 20 in PBS). Slides were permeabilised in 0.5% Triton X-100 in PBS for 15 min. Slides were washed three times for 5 min in PBST after permeabilization. After washing the slides in PBST and drawing hydrophobic rings (ImmEdge pen, H-4000, Vector), unspecific binding sites were blocked using 5% goat serum, 5% bovine serum albumin in PBST for 2 h at room temperature. Primary antibodies were incubated in blocking buffer overnight at 4°C in a humidity box. Primary antibodies used were, rabbit anti-mfap4 (AB_28867114, GTX132692, GeneTex; 1:300), rabbit anti-mpx (AB_28855768, GTX128379 GeneTex; 1:300), rabbit mCherry (AB_2571870, ab167453, Abcam; 1:500), mouse anti-zebrafish gut secretory cell epitopes (AB_1209226, ab71286, Abcam; 1:400) and chicken anti-GFP (AB_300798, ab13970, Abcam; 1:300). Slides were washed three times in PBST before incubating with secondary antibodies and DAPI as nuclear counterstaining at room temperature for 45 min. Secondary AlexaFluor-conjugated antibodies (ThermoScientific, 1:400) were used to detect isotype-specific primary antibodies and mounted with Vectashield reagent (Vector Laboratories).

### Immunofluorescence in paraffin sections

Immunofluorescence was performed as described previously (González-Rosa et al., 2011) with the following modifications. After dewaxing and rehydration, sections were heated and then pressure cooked for 4 min in citrate unmasking solution (H3300, Vector, 100× dilution). Once cooled, sections were permeabilized for 10 min in methanol, washed in PBST (0.1% Tween 20 in PBS) and photobleached for 45 min to remove autofluorescence (Lin et al., 2018). Slides were washed three times for 5 min in PBST before incubation in 0.25% Triton X-100 in PBS. After washing the slides in PBST and drawing hydrophobic rings (ImmEdge pen, H-4000, Vector), unspecific binding sites were blocked using 5% goat serum and 5% bovine serum albumin in PBST for 1 h at room temperature. Primary antibodies were incubated in blocking buffer overnight at 4°C in a humidity box. Primary antibodies used were mouse anti-RFP (AB_10999796, clone RF5R, MA5-15257 ThermoFisher; 1:1000), mouse anti-PCNA (AB_628110, SC-56, Santa Cruz Biotechnology; 1:500), rabbit anti-Lcp1 (AB_11167454, GTX124420, GeneTex; 1:500), mouse anti-col1a1 (AB_528438, clone Sp1.d8, DSHB; 1:20) and mouse anti-Anxa4 (AB_1209226, 2F11, ab71286, Abcam). Slides were washed three times for 5 min in PBST before incubation with secondary antibodies and DAPI as nuclear counterstaining in blocking buffer for 1 h at room temperature. Alexa-conjugated secondary antibodies (Life Technologies, 1:500) were used to detect isotype-specific primary antibodies, except for the detection of Col1a1, which required a tyramide amplification step (Alexa Fluor 555 Tyramide SuperBoost Kit, Invitrogen). After staining, sections were quickly rinsed in double-distilled water and mounted with Fluorsave reagent (345789, Millipore).

### Analysis of immunostained paraffin sections

After immunofluorescence, sections were imaged on a Zeiss LSM900 confocal microscope using the 10× or 20× objectives with three or four images per *z*-stack. Samples were imaged using the ZEN-pro software, and images were saved as multi-stack CZI files. Images were stacked in ImageJ (Schindelin et al., 2012), and individual channels were exported to TIF files. Multi-colour images and colour levels were adjusted using Adobe Photoshop, and figure panels were cropped and rotated to dimension in CorelDRAW.

To quantify hepatocyte proliferation, sections from *fabp10a:NLS-mKate* livers were immunostained using anti-RFP and anti-PCNA antibodies (see above). Three sections containing the largest injury areas were imaged. n-mKate^+^ and PCNA^+^n-mKate^+^ cells were counted manually using ImageJ in defined regions that include 150 µm adjacent to the border zone. The percentages of PCNA^+^n-mKate^+^/n-mKate^+^ cells from individual sections were averaged to establish the hepatocyte proliferation index for each animal.

To quantify the proliferation of the biliary epithelial cells, adjacent sections were immunostained with anti-Anxa4 and anti-PCNA antibodies (see above). Three sections containing the largest injury areas were imaged. Anxa4^+^ and PCNA^+^Anxa4^+^ cells were counted manually using ImageJ in defined regions that include 150 µm adjacent to the border zone. The percentages of PCNA^+^Anxa4^+^/ Anxa4^+^ cells from individual sections were averaged to establish the hepatocyte proliferation index for each animal.

To quantify leukocyte infiltration, sections from *fabp10a:NLS-mKate* livers were immunostained with anti-RFP and anti-Lcp1 antibodies (see above). Three sections containing the largest injury areas were imaged. The number of Lcp1^+^ cells was counted manually using ImageJ in defined regions (150 µm×150 µm) that include the injury area and border zone. Lcp1^+^ counts in three independent regions per animal were averaged to establish a quantification for each animal.

To quantify collagen deposition, liver sections were immunostained using an anti-Col1a1 antibody and imaged using confocal microscopy. Three sections containing the largest injury areas were imaged. The Col1a1^+^ area/total liver parenchyma area ratio in three independent regions per animal was averaged to establish a quantification for each animal.

### Quantification and statistical analysis

Sample sizes were chosen based on previous publications and are indicated in each figure legend. No animal or sample was excluded from the analysis unless the animal died during the procedure. The experiments were not randomized, and the investigators were not blinded to allocation during experiments and outcome assessment. All statistical values are displayed as mean±s.d. The figures or figure legends indicate sample sizes, statistical tests and *P*-values. Data distribution was determined before using parametric or non-parametric statistical tests. Statistical significance was assigned at *P*<0.05. All statistical tests were performed using Prism 7 software.

### HCR RNA-FISH staining

Samples were fixed for 24 h in 4% PFA at 4°C. Livers were washed three times for minutes in PBST (0.1% Tween 20 in PBS) on ice and permeabilised in 0.5% Triton X-100 in PBS rocking overnight. Livers were washed twice for 5 min on PBST and postfixed with 4% PFA for 20 min at room temperature. Livers were washed three times for 5 min on PBST. Samples were incubated in 1 ml probe hybridization buffer for 5 min. Afterwards, livers were incubated in 1 ml 37°C probe hybridization buffer for 30 min. Livers were incubated in 4 pmol probes overnight at 37°C with rocking. Livers were washed four times for 15 min with wash buffer at 37°C and washed again in 5×SSCT twice for 5 min. Samples were pre-amplified with 250 µl of amplification buffer for 10 min at room temperature. Samples were incubated with 6 µl of hairpin in 100 µl of amplification buffer. Excess hairpins were removed by washing twice for 5 min with 5×SSCT. Samples were nuclei counterstained using DAPI in 5×SSCT (1:1000) for 45 min, with a final wash of 15 min in 5×SSCT. Samples were mounted for imaging on a glass-bottom culture dish (MatTek Corporation) or stored at 4°C protected from light before imaging.

Two probes were designed, atf4a and fabp10a, for HCR RNA-FISH. Samples were scanned using Nikon SoRa Spinning Disk Confocal with a 10× and 20× dry lenses. Acquisitions were saved as TIFF or JPEG on Fiji of the original files.

### Bulk RNA-seq

Sham and injured livers at 1, 3 and 7 dpci were phenotyped under the fluorescent stereomicroscope (NSZ-606 Binocular Zoom fitted with a NightSea SFA light base) to confirm the presence of insult upon cryoinjury. In addition, three adult zebrafish livers were pooled per tube, discriminating between the injured border and liver tissue from other lobes. Finally, three replicates of three pooled livers were used for library preparation. Livers were transferred to a final volume of 300 μl of ice-cold TRIzol (Thermo Fisher Scientific) per tube on ice. Livers were homogenized using the mechanical homogenizer for 30 s on ice, with a plastic pestle, to ensure fine homogenization. RNA was extracted according to the manufacturer guidelines (Direct-zol RNA MiniPrep kit, Zymo Research). RNA quality was confirmed using an Agilent 4200 Tapestation System. Libraries were sequenced in Illumina NextSeq 500, with paired-end 75 bp reads to a depth of 15 M reads per sample. 7 dpf larvae bulk RNA-seq embryo liver datasets were obtained from GEO (accession number GSE195711; Vaidyanathan et al., 2022).

### Bioinformatic analysis

BCL files were converted to FastQ files on Galaxy using bcl2fastq2 (v2.20.0.422 – Illumina). Reads were mapped to the reference genome (Ensembl build 11, release 94) using Hisat2, version 2.1.0 (Kim et al., 2015), and counting was performed using featureCounts, version1.6.0 (Liao et al., 2014). For bioinformatics analysis, we compared sham-operated, 1, 3 and 7 dpci livers. Downstream analysis was performed in R, version 4.2.2 (R Core Team, 2018). Counts were normalized and differential expression between design groups was tested using the package EdgeR. Principal component analysis (PCA) plots, volcano plots and heatmaps were generated using the ggplot2 package, version 3.4.0 (Wickham 2016). For the enrichment, we selected all the Gene Stable IDs, translated them to Mus musculus Gene Stable IDs, and obtained the ENTREZIDs and SYMBOLs using biomaRt package (Durinck et al., 2005). Statistically significant genes were defined with the following thresholds, log2FC≥1.5 and *P*≤0.05. Transcriptional downstream signatures were examined using Gene Set Enrichment Analysis (GSEA v4.0.3) (Subramanian et al., 2005). Gene ontology analysis was carried out using Gene Ontology Panther (http://geneontology.org). Bulk RNA-seq data of livers collected at sham, 1 dpci, 3 dpci, and 7 dpci data have ben deposited in GEO under accession number GSE245878.

## Supplementary Material



10.1242/develop.203124_sup1 Supplementary information

Table S1. Bulk RNA-seq of normalised counts following cryoinjury
