# Supplementary material for: Development of a hepatic cryoinjury model to study liver regeneration
Source: Development. 2024 Jul 31;151(15):dev203124. doi: 10.1242/dev.203124 (PMC11318111; doi:10.1242/dev.203124)
Supplement: Supplementary file 1 [file develop-151-203124_review_history.pdf]

Development of a hepatic cryoinjury model to study liver regeneration

Marcos Sande-Melon, David Bergemann, Miriam Fernández-Lajarín, Juan M González-Rosa and Andrew Graham Cox
DOI: 10.1242/dev.203124

Editor: James Wells

Review timeline

Submission to Review Commons	7 August 2023
Submission to Development:	29 May 2024
Accepted:	20 June 2024

Reviewer 1

Evidence, reproducibility and clarity

In this manuscript titled "Development of a hepatic cryoinjury model to study liver regeneration" by Sande-Melon et al., the authors developed a novel model to study liver regeneration, namely a cryoinjury model in adult zebrafish. The authors described the methodology in detail and extensively characterized the kinetics of liver regeneration in this model, including hepatocyte necrosis/apoptosis, the proliferation of hepatocytes, cholangiocytes, endothelial cells, and infiltration of leukocytes. Most of the characterization were performed by immunostaining for various cell markers, which the authors corroborated with transcriptomic analysis by bulk RNAseq.

Major comments:

- While the authors assayed changes in major cell types during liver regeneration in this model, the selection of varying timepoints for analysis and incomplete quantification for all timepoints precludes detailed comparisons that may lead to mechanistic insights. For example, closure of injury area is assayed at 1,3,7,14 dpci but hepatocyte proliferation is measured at 1,3,5,7, 18, 30 dpi. Fibrosis was only assayed at 5 dpi (assume dpi is the same as dpci). Cholangiocytes and endothelial cells are imaged at 1, 3, 7, 30 dpci but no quantification was provided only a single image. Since most changes are occurring at 1-7 dpci, the authors should at least measure the same timepoints from 1-7 dpci for the different cell types so comparisons can be made and conclusions can be drawn. For example, does hepatocyte proliferation, which seem to peak at 5 dpci, happen before endothelial proliferation, which is measured at 3 and 5 dpci but not measured at 5 dpci?
- Fibrosis level seems to be highly variable at 5dpci, which is the only time point measured. If this level of variability is found across all timepoints then this might not be a good model to study the intersection of fibrosis and regeneration. Since the authors have collected animals at all timepoints, it should be fairly straight forward to carry out collagen staining and quantification across different timepoints without the need of additional fish experiments.
- The lack of quantification of cholangiocytes and endothelial cells makes it difficult to gauge the reproducibility of this model across different animals and experiments.
- Transcriptomic data analysis/presentation in Figure 7 can be improved. Cannot read any of the gene labels in Figure 7B. Figure 7H should use at least a few different gene markers from each cell type to approximate cell abundance.
- OPTIONAL: Sheets of DAPI staining are observed in Figure 6G'. Is this DNA from necrotic cells? Could they make up a neutrophil extracellular trap (NET)-scaffold like structure that covers/protects the injury site from infection? This is purely speculative but might represent an interesting area of study.

© 2023. Published by The Company of Biologists under the terms of the Creative Commons Attribution License (https://creativecommons.org/licenses/by/4.0/).

- OPTIONAL: To demonstrate this a useful model that complements existing models of liver regeneration, the authors can try to capitalize on the proposed strength of the model to provide some novel insights into liver regeneration. A notable feature of this model that is missing from the PHx and APAP rodent models is the development of robust fibrosis that rapidly resolves within a short time frame, providing an unique opportunity to investigate the potential crosstalk between fibrosis and regeneration that often co-occur in chronic liver disease patients.

Minor comments:

- Is "dpi" the same as "dpci"? Please use the same nomenclature throughout manuscript
- In the mouse PHx model, hepatocytes reach max proliferation (as measured with Ki67/PCNA staining) at 40-48hrs across different labs and experiments, not at 24rs
- Zebrafish references are used when the author is talking about mouse PHx model on page 12

Significance

Mouse 2/3 partial hepatectomy surgery (PHx) is the most frequently used model to study liver regeneration and much has been learnt from this model. However, mouse PHx involving tying off certain lobes of the liver and the inducing a sterile injury, where hepatocyte proliferation and liver regeneration occurs in the absence of significant inflammation and fibrosis. To understand the full complexity of the liver regeneration response, especially against the backdrop of a necroinflammatory environment that characterize chronic liver disease in patients, alternative models to study liver regeneration have been used such as the rodent APAP model of chemically induced injury. Here, Sande-Melon et al. aims to establish such a liver regeneration model in adult zebrafish that would harness the power of the zebrafish model, such as availability of various transgenic lines that label different cell populations, ease of accessibility to imaging techniques, large N number, and the convenience of working with lower complexity model organisms. While such a zebrafish liver regeneration model will be welcomed by the greater research community interested in studying liver regeneration, this paper in its current forms falls short of demonstrating the robustness and reproducibility of this model that would make it a useful research tool.

Reviewer 2

Evidence, reproducibility and clarity

In this manuscript, Sande-Melon et al described a new model for studying liver regeneration in zebrafish that is induced by cryoinjury. They showed that this model induced hepatocyte proliferation, transient fibrosis and inflammation, and regeneration of the biliary and vascular network. Compared to the other established models, such as partial hepatectomy, drug-induced liver injury, the cryoinjury model is easy to perform, consistent, and involves shorter recovery time. Overall, it is a useful tool that complements existing liver regeneration models. The tissue clearing methodology is highly effective.

Main critiques:

1. It is not clear whether both male and female fish were used in the analyses and whether there is any gender difference in regeneration responses at cellular and molecular levels. The method mentioned that 4-9 month old fish were used in the study. Was there any difference between young and old fish?
2. The authors detected increased hepatocyte proliferation following cryoinjury. It will be interesting to investigate if activation of stem cells and transdifferentiation of cholangiocytes also contribute to regeneration in this particular model.
3. It will be important to characterize hepatic stellate cells, macrophages, and neutrophils in this model, given their critical and complex roles in liver regeneration. Transgenic reporter lines marking these cells types are available.
4. It is not appropriate to call Fli1a + cells liver sinusoidal cells. As far as I know, there is no specific marker for LSEC in zebrafish. Fli1a transgene labels all vascular cells.

Significance

© 2023. Published by The Company of Biologists under the terms of the Creative Commons Attribution License (https://creativecommons.org/licenses/by/4.0/).

In this manuscript, Sande-Melon et al described a new model for studying liver regeneration in zebrafish that is induced by cryoinjury. They showed that this model induced hepatocyte proliferation, transient fibrosis and inflammation, and regeneration of the biliary and vascular network. Compared to the other established models, such as partial hepatectomy, drug-induced liver injury, the cryoinjury model is easy to perform, consistent, and involves shorter recovery time. Overall, it is a useful tool that complements existing liver regeneration models. The tissue clearing methodology is highly effective.

Reviewer 3

Evidence, reproducibility and clarity

Summary:

In this study, the authors presented a novel cryoinjury model of liver damage and regeneration that reflects essential features of liver disease, including local fibrosis. Because of its rapid and consistent method, this model will be helpful and provide opportunities to delve into the molecular basis of liver regeneration. This manuscript also contains a high technique of visualization of the regenerating liver. The manuscript is well-written, and the points are clear. However, this form of manuscript might be overly descriptive, and adding functional, mechanical, or lineage tracing-based fate decision insights would make this manuscript significantly better.

Major points:

1. In this cryoinjury model, the authors found cell proliferation in hepatocytes, BECs, and other cell types near the injury site. The proliferating hepatocytes exclusively provide hepatocytes, and BECs provide BECs, or some transdifferentiation is involved? Like other extreme ablation models, BECs can contribute to some hepatocytes in this model.
2. In this model, the authors observed the long-range effect of the cryoinjury as they identified increased cell proliferation in the contralateral liver lobes. Is this long-range effect specific to hepatocytes? BECs or endothelial cells also undergo increased cell proliferation in the contralateral lobes?
3. This model is a unique liver regeneration model as it induces transient focal fibrosis. Is the fibrosis beneficial for liver regeneration? What happens if you reduce fibrosis pharmacologically? Will it interfere with the rate of regeneration?
4. Do Lcp1+ leucocytes contribute to liver regeneration in this mode? In immunodeficiency models such as irf8 mutant, liver regeneration after cryoinjury changed?
5. The CUBIC-clearing procedure is beneficial in the field. The quantitative benefit of the CUBIC-based method should be added. Supplement figures 1C and D need scale bars, especially for X-Z and Z-Y planes. Can you quantify the Z-plane depth that you can scan with or without CUBIC treatment?
6. In the manuscript, the authors measured the injured area after the cryoinjury. But how about the depth of the injury? Does the procedure induce a relatively constant injury depth, or can it not be controlled? The total volume of injured tissue would be more important than the surface injured area.

Minor points:

7. The sham procedure means exposing the liver by removing the scale and cutting the skin, right? What is the survival rate of the sham procedure? Is the survival rate of sham group significantly lower than cryoinjury-induced group?
8. The original RNA-seq data, including FASTQ files, should be deposited to NCBI (Gene Expression Omnibus) or other public databases.

Significance

The strength of this manuscript is that the authors established the new cryoinjury liver regeneration model. Compared to other models, this model introduced local fibrosis and relatively

© 2023. Published by The Company of Biologists under the terms of the Creative Commons Attribution License (https://creativecommons.org/licenses/by/4.0/).

quick resolution of the fibrosis, which is unique to this model. Fibrosis is like a double-edged sword, as it can be a severe problem, but it may also enhance healing and regeneration. This useful model would advance our understanding of the role of fibrosis in liver regeneration. Also, this manuscript contains important new technologies, such as CUBIC-clearing, and will be helpful for the research field.

Author response to reviewers' comments

Manuscript number: RC- 2023-02122

Corresponding author(s): Andrew Graham Cox and Juan Manuel González-Rosa

1. General Statements

We thank the reviewers for taking the time to assess our work and for their considered and constructive comments. We are glad that they appreciate the value of the methodology we have developed. In addressing the points raised by the reviewers, we have significantly strengthened the conclusions reached in our study. Below is a point-by-point response (in regular type, blue) to the specific reviewer comments (in italics, black).

2. Point-by-point description of the revisions

Experiment 1: Perform lineage tracing of hepatocytes following cryoinjury.
Reviewer #1 would like us to have a better understanding of the origin of the regenerative hepatocytes following cryoinjury. There are two potential sources of regenerating hepatocytes. In many cases, hepatocytes proliferate giving rise to regenerative hepatocytes. However, during severe injury, the liver can undergo a ductular reaction in which biliary epithelial cells (BECs) can expand and transdifferentiate to give rise to regenerating hepatocytes.

- To address this query we have now used a new transgenic line created in laboratory that can indelibly label hepatocytes for lineage tracing Tg(*fabp10a:Tet-ON-Cre)*. We have crossed this line to floxed reporters *(Ubb:Switch)* and collect livers at 7 dpci. The healthy parenchyma surrounding the injured area was predominantly labelled in the tracing experiment suggesting that pre-existing hepatocytes are driving the regenerative response.

Experiment 2: Examine BEC and EC proliferation in the ventral and contralateral lobes following cryoinjury.
Reviewers #1 and #2 would like us to better characterise the temporal dynamics of proliferation in BECs and ECs following cryoinjury. Specifically, the reviewers would like to know whether then compensatory hyperplasia in the contralateral lobe also leads to increased BEC and EC proliferation. Moreover, the reviewers would like us to better quantify the extent of EC and BEC proliferation at different stages of regeneration after cryoinjury.

- We have now performed extensive BrdU pulse-chase cryoinjury experiments using Tg(*fli1a:nEGFP*) zebrafish to visualise ECs. We have also conducted multiplexed immunostaining of the regenerating livers with the BEC marker (Anxa4) in conjunction with immunodetection of proliferation (BrdU and PCNA). These studies outline the kinetics of the regenerative response and provide evidence to support epimorphic regeneration around the site of injury as well as a compensatory hyperplasia on the contralateral lobe.

Experiment 3: Quantification of the temporal dynamics of fibrosis upon cryoinjury.
Reviewer #1 suggested we better characterise the extent of fibrosis in our model.

- We have now performed extensive studies quantifying the extent of collagen deposition at the regenerative margin over the time course (SHAM, 1, 3, 5, and 7 dpci) using immunohistochemical detection.

© 2023. Published by The Company of Biologists under the terms of the Creative Commons Attribution License (https://creativecommons.org/licenses/by/4.0/).

Experiment 4: Examine the role of Macrophage depletion in liver regeneration.
Reviewer #1 suggested we examine regeneration following cryoinjury in immunodeficient zebrafish in order to understand the role of macrophages in the model.

- To address this question, we have now performed studies involving macrophage depletion, using the well established IP injection of clodronate liposomes. We have now performed cryoinjury comparing untreated and chlodronate-treated Tg(*fabp10a:NLS- mCherry)* or Tg(*fabp10a: GreenLantern-H2B*) zebrafish and examined the extent of regeneration at 3 and 7 dpci.

Experiment 5: Examine the impact of age and gender on liver regeneration following cryoinjury.
Reviewer #3 wanted to know if the regenerative response to cryoinjury was different depending on age and gender.

- To address this query, we have now performed cryoinjuries on young (4 month) and aged (9 month) males and females in a Tg(f*abp10a:NLS-mCherry*) or Tg(*fabp10a: GreenLantern-H2B*) background and examined regeneration at 7 dpci.

Experiment 6: Characterization of the dynamics of Hepatoblasts, Hepatic Stellate Cells, Macrophages and Neutrophils following cryoinjury.
Reviewer #3 suggested that it would be good to have a better cellular characterization of regeneration in the cryoinjury model.

- To address this question, we have now examined distinct cell types over the cryoinjury timecourse including SHAM, 1, 3, 5, and 7 dpci livers to provide a temporal landscape of the cellular response. In addition to BECs and ECs as discussed above, we have also performed immunofluorescence to detect macrophages (mfap4) neutrophils (mpx) during liver regeneration.

<u>Specific Reviewer comments</u>
Reviewer #1
Major points:
1) In this cryoinjury model, the authors found cell proliferation in hepatocytes, BECs, and other cell types near the injury site. The proliferating hepatocytes exclusively provide hepatocytes, and BECs provide BECs, or some transdifferentiation is involved? Like other extreme ablation models, BECs can contribute to some hepatocytes in this model.
We thank the Reviewer #1 for the interesting suggestion. We have addressed this by performing lineage tracing analysis as explained in Experiment 1 (above). For this approach, we have used Tg(*fabp10a:Tet-ON-Cre; Ubb:Switch*) to indelibly label and trace hepatocytes. These experiments reveal that the new regenerated tissue is derived from pre-existing hepatocytes (see Supplementary Figure 2 Q, R, S, T).

2) In this model, the authors observed the long-range effect of the cryoinjury as they identified increased cell proliferation in the contralateral liver lobes. Is this long-range effect specific to hepatocytes? BECs or endothelial cells also undergo increased cell proliferation in the contralateral lobes?
We thank the Reviewer #1 for this question. We have addressed this query by performing Experiment 2 (above). Briefly, cryoinjuries were performed and markers of proliferating HCs and BECs (PCNA or BrdU stained) were quantified in the ventral and contralateral lobes (see Supplementary Figure 6). The data clearly demonstrates that proliferation is higher at the site of injury, however lower rates of compensatory hyperplasia are still evident on the contralateral lobe. A strong epimorphic hyperplasia and weaker compensatory growth response, has been previously observed in the cardiac cryoinjury model (Pauline Sallin *et al*. Developmental Biology 2015).

3) This model is a unique liver regeneration model as it induces transient focal fibrosis. Is the fibrosis beneficial for liver regeneration? What happens if you reduce fibrosis pharmacologically? Will it interfere with the rate of regeneration?
We thank the Reviewer #1 for the comments. Although pharmacological interventions of fibrosis are beyond the scope of the current manuscript, we have better quantified the extent of fibrosis in the first week following cryoinjury in Experiment 3 (above; Figure 3I).

© 2023. Published by The Company of Biologists under the terms of the Creative Commons Attribution License (https://creativecommons.org/licenses/by/4.0/).

4) Do Lcp1+ leucocytes contribute to liver regeneration in this model? In immunodeficiency models such as irf8 mutant, liver regeneration after cryoinjury changed?
We thank the Reviewer #1 for the suggestion of using an immunodeficiency model. We addressed this question by performing Experiment 4 (above). Briefly, we have IP injected clodronate liposomes, which are a well-established method for macrophage depletion, and examined the effect on liver regeneration (Supplementary Figure 5). These extensive experiments showed that macrophage depletion had no significant effect on liver regeneration at 3 and 7 dpci.

5) The CUBIC-clearing procedure is beneficial in the field. The quantitative benefit of the CUBIC-based method should be added. Supplement figures 1C and D need scale bars, especially for X-Z and Z-Y planes. Can you quantify the Z-plane depth that you can scan with or without CUBIC treatment?
We thank the Reviewer #1 for the input and apologise if we did not present the current data clearly. We have now included the scale bars on the reviewed manuscript in Supplementary Figure 1C, 1D, and 1G. We have quantified the Z-plane depth on our current acquisitions and modified our current panels to make clear the difference in depth (z-stack) that CUBIC-imaging enables during liver acquisitions in Supplementary Figure 1D-I.

6) In the manuscript, the authors measured the injured area after the cryoinjury. But how about the depth of the injury? Does the procedure induce a relatively constant injury depth, or can it not be controlled? The total volume of injured tissue would be more important than the surface injured area.
We thank the Reviewer #1 for the comments. The hepatic cryoinjury approach was developed to injure the liver and avoid deeper tissue lesions to the gastrointestinal tract. Our existing CUBIC data suggests that injury depth remains constant.

Minor points:
7) The sham procedure means exposing the liver by removing the scale and cutting the skin, right? What is the survival rate of the sham procedure? Is the survival rate of sham group significantly lower than cryoinjury-induced group?
The Reviewer #1 is correct about the cryoinjury procedure in SHAM samples. SHAM survival is 95% while the injured animal survival is 92.97% (Figure below; n= 444). This analysis shows no significant difference between the groups (unpaired Student's t-test; p-value: 0.5843)

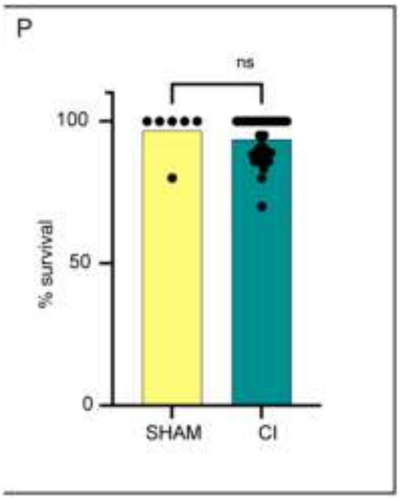

8) The original RNA-seq data, including FASTQ files, should be deposited to NCBI (Gene Expression Omnibus) or other public databases.
We apologize for not submitting our Bulk RNA-seq data to NCBI GEO during the initial submission. The Bulk RNA-seq data can be found under the accession number GSE245878.

Reviewer #2
Major points:

© 2023. Published by The Company of Biologists under the terms of the Creative Commons Attribution License (https://creativecommons.org/licenses/by/4.0/).

1) While the authors assayed changes in major cell types during liver regeneration in this model, the selection of varying timepoints for analysis and incomplete quantification for all timepoints precludes detailed comparisons that may lead to mechanistic insights. For example, closure of injury area is assayed at 1,3,7,14 dpci but hepatocyte proliferation is measured at 1,3,5,7, 18, 30 dpi. Fibrosis was only assayed at 5 dpi (assume dpi is the same as dpci). Cholangiocytes and endothelial cells are imaged at 1, 3, 7, 30 dpci but no quantification was provided only a single image. Since most changes are occurring at 1-7 dpci, the authors should at least measure the same timepoints from 1-7 dpci for the different cell types so comparisons can be made and conclusions can be drawn. For example, does hepatocyte proliferation, which seem to peak at 5 dpci, happen before endothelial proliferation, which is measured at 3 and 5 dpci but not measured at 5 dpci?
We thank the Reviewer #2 for the comments regarding temporal dynamics of regeneration. In response we have performed Experiment 2 (above). Briefly, this included examination of BECs and ECs at different time points during regeneration (SHAM, 1, 3, 5, and 7 dpci; Figure 6, Supplementary Figure 6L-P).

2) Fibrosis level seems to be highly variable at 5 dpci, which is the only time point measured. If this level of variability is found across all timepoints then this might not be a good model to study the intersection of fibrosis and regeneration. Since the authors have collected animals at all timepoints, it should be fairly straight forward to carry out collagen staining and quantification across different timepoints without the need of additional fish experiments.
We thank the Reviewer #2 for the comments regarding the fibrotic response. In response we have undertaken Experiment 3 (above). This experiment involves quantifying collagen deposition at the different timepoints (SHAM, 1, 3, 5, and 7 dpci; Figure 3I).

3) The lack of quantification of cholangiocytes and endothelial cells makes it difficult to gauge the reproducibility of this model across different animals and experiments.
We thank the Reviewer #2 for the comments regarding the need to quantify ECs and BECs during regeneration. In response we will undertake Experiment 2 (above). Briefly, this included examination of BECs and ECs at different time points during regeneration (SHAM, 1, 3, and 7 dpci; Figure 6 and Supplementary Figure 7).

4) Transcriptomic data analysis/presentation in Figure 7 can be improved. Cannot read any of the gene labels in Figure 7B. Figure 7H should use at least a few different gene markers from each cell type to approximate cell abundance.
We apologise for the inconvenience and have addressed the issue of legibility. We have increased font size on the volcano plots in Figure 7 and incorporate a new analysis with more markers for each cell type in Figure 7H. In addition, we have included the comparison between Bulk RNA-seq ventral samples and contralateral lobe samples, together with further GO- enrichment of the samples in Supplementary Figure 8.

Minor:

5) Is "dpi" the same as "dpci"? Please use the same nomenclature throughout manuscript.
We apologise. Dpi means days post-injury and dpci means days post-cryoinjury. Nomenclature has been corrected in revised version of the manuscript.

6) In the mouse PHx model, hepatocytes reach max proliferation (as measured with Ki67/PCNA staining) at 40-48hrs across different labs and experiments, not at 24rs.
We thank the Reviewer #2, we have changed this reference.

7) Zebrafish references are used when the author is talking about mouse PHx model on page 12.
We thank the Reviewer #2, we have changed this reference 7 and 8 to reference the right papers.

Reviewer #3
Major points:

1) It is not clear whether both male and female fish were used in the analyses and whether there is any gender difference in regeneration responses at cellular and molecular levels. The method mentioned that 4-9 month old fish were used in the study. Was there any difference between young and old fish?

© 2023. Published by The Company of Biologists under the terms of the Creative Commons Attribution License (https://creativecommons.org/licenses/by/4.0/).

We thank the Reviewer #3 for the comments regarding the need to consider age and gender in regeneration studies. Our experiments have been performed on adult male zebrafish. To examine the impact of age and gender on regeneration we have performed Experiment 5 (above). In brief, we have undertaken cryoinjuries in 4 month or 9 month old females and males in the Tg(f*abp10a:NLS-mCherry*) or Tg(f*abp10a: GreenLantern-H2B*) background and examine regeneration at 7 dpci (Supplementary Figure 2 J-N and P. We could not detect a significant difference among any of these comparisons. However, we observed a subtle trend with female adult zebrafish showing smaller insult area compared to adult male zebrafish, both at 3 and 7 dpci (Supplementary Figure 2P).

2) The authors detected increased hepatocyte proliferation following cryoinjury. It will be interesting to investigate if activation of stem cells and transdifferentiation of cholangiocytes also contribute to regeneration in this particular model.
We thank the Reviewer #3 for the comments regarding the need to examine the potential involvement of hepatoblasts and transdifferentiating BECs in regeneration following cryoinjury. We have addressed these aspects with Experiment 6 (above). Briefly, we have performed cryoinjuries in adult zebrafish and utilised Anxa4 staining for detection of BECs at SHAM, 1, 3, 5, and 7 dpci (Figure 6A-F). This analysis showed that the were no detectable signs of transdifferentiation between hepatocytes and cholangiocytes (ie: there were no double positive cells ($Anxa^{+}$/*fabp10a:H2B-GreenLantern*$^{+}$ or *fabp10a:H2B-mCherry*$^{+}$). Moreover, we performed lineage tracing experiments and found evidence that pre-existing hepatocytes give rise to the regenerating tissue (Supplementary Figure 2 Q-T). Together, these experiments indicate that hepatocytes are responsible for the regeneration of the liver upon cryoinjury without the necessity of BEC transdifferentiation.

3) It will be important to characterize hepatic stellate cells, macrophages, and neutrophils in this model, given their critical and complex roles in liver regeneration. Transgenic reporter lines marking these cell types are available.
We thank the Reviewer #3 for the comments regarding the need to examine hepatic stellate cells (HSCs), macrophages and neutrophils in regeneration following cryoinjury. We have addressed these aspects with Experiment 6 (above). Briefly, we have studied the temporal dynamics of neutrophils upon cryoinjury by immunofluorescent detection of myeloperoxidase (mpx) (Supplementary Figure 4). We have also explored the role of macrophage depletion in response to cryoinjury by performing clodronate injections. We found no significant changes in liver regeneration following clodronate injections (Supplementary Figure 5). To examine the temporal dynamics of HSCs we attempted to use two approaches, namely imaging transgenic lines labelling HSCs (Tg(*BAC-pdgfrb:EGFP*) and HCR for HSCs (*pdgfrb*), but unfortunately we were not able to detect HSCs with these approaches.

4) It is not appropriate to call Fli1a + cells liver sinusoidal cells. As far as I know, there is no specific marker for LSEC in zebrafish. Fli1a transgene labels all vascular cells.
We acknowledge this mistaken nomenclature and have made the necessary amendment to use the term endothelial cells (ECs).

Original submission

First decision letter

MS ID#: DEVELOP/2024/203124

MS TITLE: Development of a hepatic cryoinjury model to study liver regeneration

AUTHORS: Marcos Sande-Melon, David Bergemann, Miriam Fernández-Lajarín, Juan M González-Rosa, and Andrew Graham Cox
ARTICLE TYPE: Research Article

Thank you for sending your manuscript to Development through Review Commons.

© 2023. Published by The Company of Biologists under the terms of the Creative Commons Attribution License (https://creativecommons.org/licenses/by/4.0/).

I am happy to tell you that your manuscript has been accepted for publication in Development, pending our standard publication integrity checks.

Reviewer 1

Advance summary and potential significance to field

Mouse 2/3 partial hepatectomy surgery (PHx) is the most frequently used model to study liver regeneration and much has been learnt from this model. However, mouse PHx involving tying off certain lobes of the liver and the inducing a sterile injury, where hepatocyte proliferation and liver regeneration occurs in the absence of significant inflammation and fibrosis. To understand the full complexity of the liver regeneration response, especially against the backdrop of a necroinflammatory environment that characterize chronic liver disease in patients, alternative models to study liver regeneration have been used such as the rodent APAP model of chemically induced injury. Here, Sande-Melon et al. aims to establish such a liver regeneration model in adult zebrafish that would harness the power of the zebrafish model, such as availability of various transgenic lines that label different cell populations, ease of accessibility to imaging techniques, large N number, and the convenience of working with lower complexity model organisms. While such a zebrafish liver regeneration model will be welcomed by the greater research community interested in studying liver regeneration, this paper in its current forms falls short of demonstrating the robustness and reproducibility of this model that would make it a useful research tool.

Comments for the author

The authors have satisfactorily addressed all my previous comments in the revised manuscript.

Reviewer 2

Advance summary and potential significance to field

The authors performed additional experiments to address my previous comments.

Comments for the author

I have two comments on the new data.
1. Potential contribution of biliary cell transdifferentiation to regeneration. Both Figure 6A-F and Fig S2 Q-T are of insufficient resolution. It is not possible to tell whether this is colocalization at cellular level. To demonstrate there is no transdifferentiation of biliary cells to hepatocytes, one should use the Tp1:CreERT2 line to lineage trace the biliary cells during regeneration.
2. Clodronate injections. It is not clear when clodronate injection was performed and how quickly it ablated the macrophages. The earliest time point shown in Fig S4 is 3 dpi. Macrophage may contribute to liver regeneration prior to that.

Reviewer 3

Advance summary and potential significance to field

In this study, the authors developed a new cryoinjury-based model of liver regeneration. There are several models of liver regeneration in zebrafish, but this is the only one that consistently induces fibrosis during regeneration. In the human liver, generation is frequently associated with fibrosis, and there is no good established liver fibrosis model that is resolved in time in zebrafish; this new model will be an excellent model.

Comments for the author

The authors have addressed the concerns that I raised before, and I have no further questions.

© 2023. Published by The Company of Biologists under the terms of the Creative Commons Attribution License (https://creativecommons.org/licenses/by/4.0/).
